# Release of sputum neutrophil granules is associated with pulmonary function and disease severity in childhood asthma

**DOI:** 10.1186/s12890-024-03340-y

**Published:** 2024-10-24

**Authors:** Min Jung Kim, Soo Yeon Kim, Jong Deok Kim, Mireu Park, Yoon Hee Kim, Kyung Won Kim, Myung Hyun Sohn

**Affiliations:** 1 Department of Pediatrics, Yongin Severance Hospital, Yonsei University College of Medicine, Yongin- si, Gyeonggi-do Korea; 2 Department of Pediatrics, Severance Hospital, Institute of Allergy, Brain Korea 21 PLUS Project for Medical Science, Yonsei University College of Medicine, 50-1 Yonsei-Ro, Seodaemun-gu, Seoul, 03722 Korea; 3 Department of Pediatrics, Gangnam Severance Hospital, Yonsei University College of Medicine, Seoul, Korea

**Keywords:** Asthma, Children, Induced sputum, Neutrophils, Pulmonary function

## Abstract

**Background:**

Myeloperoxidase (MPO) and human neutrophil lipocalin or neutrophil gelatinase-associated lipocalin (HNL/NGAL) are stored in neutrophil granulocytes and secreted upon activation of the cells. They have been proposed to reflect the degree of inflammation in the airways. However, their role as potential markers of disease severity in childhood asthma remains unknown. This study investigated the relationship between the expression of MPO and HNL/NGAL and childhood asthma.

**Methods:**

A total of 83 pediatric patients with asthma and 59 controls were enrolled. Using enzyme-linked immunosorbent assays, the human MPO and HNL/NGAL levels were measured in sputum supernatants. Assessments including spirometry, methacholine challenge test, and atopy test were conducted.

**Results:**

No difference in sputum neutrophil counts was observed between pediatric patients with asthma and controls. However, sputum MPO and HNL/NGAL levels were significantly higher in patients with asthma than in controls (*p* = 0.021 and *p* < 0.001, respectively), especially in patients with moderate-to-severe persistent asthma. In patients with asthma, sputum MPO and HNL/NGAL levels showed a positive correlation with sputum neutrophil counts (MPO, *r* = 0.433, *p* < 0.001; HNL/NGAL, *r* = 0.584, *p* < 0.001) and with each other (*r* = 0.628, *p* < 0.001). Moreover, sputum HNL/NGAL level demonstrated better ability to accurately reflect current pulmonary function, airway inflammation, and limitations than MPO level in this study.

**Conclusions:**

Sputum MPO and HNL/NGAL levels, which reflect neutrophil activation in airways, were increased in pediatric patients with asthma. Moreover, sputum MPO and HNL/NGAL may serve as appropriate assessment indicators of asthma severity in pediatric patients.

**Supplementary Information:**

The online version contains supplementary material available at 10.1186/s12890-024-03340-y.

## Background

Eosinophils and type 2 inflammations (T2) have long been thought to play a central role in the development of asthma, contributing to airway hyperresponsiveness, variable airway obstruction, and airway remodeling [1]. However, recent investigations into asthma phenotypes have highlighted the significance of eosinophil-low and T2-low types of noneosinophilic inflammation [2–4].

The prevalence of noneosinophilic airway inflammation may vary among studies owing to different cutoff values of sputum cell counts [5]. Nonetheless, noneosinophilic airway inflammation, including neutrophilic and paucigranulocytic phenotypes, occurs in more than 50% of patients with asthma [5, 6]. Previous cluster analyses performed on large populations have reported association between sputum neutrophil counts and severe asthma phenotypes [1, 2]. Sputum neutrophilia has been found in up to 80% of patients with asthma exacerbation [5]. It is frequently observed in children with asthma and are considered crucial for phenotypic assessment [7]. Although the precise mechanism of interaction between neutrophils and asthma is still under investigation, various studies have suggested the role of neutrophils in asthma pathogenesis [5–8].

Neutrophils contain several proteins localized in their granules, including potent antimicrobial peptides and proteolytic enzymes specific to the neutrophils. They are expressed in several tissues, constitutively or in response to stimulation by proinflammatory mediators. Among many neutrophil granules, myeloperoxidase (MPO) [9] and human neutrophil lipocalin or neutrophil gelatinase-associated lipocalin (HNL/NGAL) [10] have been proposed as cell-specific markers for neutrophils in sputum and bronchoalveolar lavage fluids. Elevated MPO and HNL/NGAL levels in body fluids or tissues have been reported in various airway diseases [11–14].

To better understand the association between neutrophils and childhood asthma, we characterized the expression of MPO and HNL/NGAL levels in pediatric patients with asthma and investigated whether those could reflect airway inflammation and pulmonary function in childhood asthma.

## Methods

### Participants

In this study, we enrolled 142 pediatric patients aged 5–18 years who visited Severance Children’s Hospital between January 2015 and December 2019. Among the 142 pediatric patients, 83 were diagnosed with asthma, defined as recurrent wheezing or coughs in the absence of a cold in the preceding 12 months with a physician’s diagnosis and bronchial hyperresponsiveness (BHR) upon methacholine challenge (PC_20_ ≤ 16 mg/mL) or at least 12% reversibility of forced expiratory volume in 1 s (FEV_1_) after inhalation of a bronchodilator (BD) [15]. Following the 2022 Global Initiative for Asthma guidelines, the severity of asthma and level of control were evaluated. Pediatric patients with a history of acute respiratory infection or asthma exacerbation in the preceding 4 weeks were excluded from the study. Patients with asthma were subcategorized based on the severity of asthma into intermittent, mild persistent, and moderate-to-severe groups (15, 29, and 39 patients in each group, respectively).

The control group consisted of 59 children who had visited the hospital for general health workups or vaccinations and had no history of wheezing, recurrent or chronic diseases, infection in the preceding 4 weeks, or BHR to methacholine.

Total serum immunoglobulin E (IgE) levels and peripheral blood cell counts were measured for all patients at the initiation of the evaluation. Specific IgE levels to six common allergens in Korea were analyzed. Atopy was defined as specific IgE level of > 0.7 KUa/L for more than one allergen, total IgE level of > 150 IU/mL, or positive skin test reactions to 12 common aeroallergens [16, 17].

This study was approved by the Institutional Review Board of Severance Hospital (Seoul, Korea) (protocol no. 4-2024-0366). Written consent for participation was obtained from parents, with verbal permission from the pediatric patients.

### Spirometry with impulse oscillometry (IOS) and methacholine challenge test

Spirometry (Jaeger Co., Würzburg, Germany) was performed, and flow volumes were calculated according to the American Thoracic Society guidelines before and after BD inhalation [18]. IOS was performed using a Jaeger MasterScreen IOS system (Jaeger Co.) under standard maneuvers. The impulse generator produced brief pressure pulses at intervals of 0.2 s [19, 20]. IOS parameters included the mean respiratory resistance at 5 Hz (R5) and 10 Hz (R10) and the difference between respiratory resistance at 5 and 20 Hz (R5-20). R5 reflects obstruction in the total airways, R20 only reflects obstruction in the large airways, and the difference between R5 and R20 (R5-20) is a parameter of the small airway. Reactance values at 5 Hz (X5) and reactance area (AX) were also recorded, which reflect the degree of obstruction in the peripheral airways [19, 20].

The methacholine challenge test was performed according to standardized procedures [21]. Each child inhaled increasing concentrations of methacholine (0.075, 0.15, 0.31, 0.62, 1.25, 2.5, 5, 10, 25, and 50 mg/mL) nebulized by a dosimeter (MB3; Mefar, Brescia, Italy) until the FEV_1_ reduced by 20% from a post-nebulized saline solution value. The bronchial response was expressed as a provocative concentration of methacholine, causing a 20% fall in FEV_1_ (PC_20_).

### Fractional concentration of exhaled nitric oxide (FeNO) measurement

FeNO was measured using a CLD 88 (Eco Medics, Duernten, Switzerland) at a constant expiratory flow rate of 50 mL/s. The measurements were conducted according to standard guidelines [22]. Because FeNO levels could be affected by nitrate-rich foods, all patients refrained from eating nitrate-rich foods for 2 h before FeNO level measurement. The mean value of the three consecutive measurements was calculated and regarded as the actual value [19, 20].

### Sputum induction and processing

Sputum induction and processing were performed as previously described [23]. Sputum samples were kept at 4 °C for less than 2 h before further processing. Sputum viability was determined by the trypan blue exclusion method. Total cell counts were performed with a hemocytometer, and slides were prepared with cytospin (Cytospin3; Shandon, Tokyo, Japan) and stained with May–Grünwald–Giemsa stain. An adequate specimen was defined as one producing countable cytospin slides to estimate differential cell count with minimal squamous contamination (< 50%) [20]. Differential cell counts were performed by two observers who were blind to clinical details, analyzing 400 non-squamous cells. Sputum cell numbers were recorded as percentages (%) for each cell type. Sputum inflammatory subtypes were classified as follows: eosinophilic (sputum eosinophils ≥ 3% and sputum neutrophils < 61%), mixed (sputum eosinophils ≥ 3% and sputum neutrophils ≥ 61%), neutrophilic (sputum eosinophils < 3% and sputum neutrophils ≥ 61%), and paucigranulocytic (sputum eosinophils < 3% and neutrophils < 61%) [24, 25]. Sputum supernatants were stored at − 70 °C until the following experiments.

### Measurement of MPO and HNL/NGAL in sputum

According to the manufacturer’s instructions, human MPO and HNL/NGAL were individually detected using enzyme-linked immunosorbent assay kits (R&D Systems, Minneapolis, MN, USA). The lower detection limit of assays was 0.062 ng/mL for MPO and 0.04 ng/mL for HNL/NGAL.

### Statistical analysis

Numerical variables with normal distribution were expressed as the mean and standard deviation. Normal distribution was determined by Kolmogorov–Smirnov test. Numerical parameters with non-normal distribution were presented as the median and interquartile range. The Mann–Whitney U test was used for the statistical comparison of values between the groups. The correlation between sputum MPO and HNL/NGAL levels and numerical parameters was determined using the Spearman’s rank correlation test. All comparisons were two-sided. A *p*-value < 0.05 was considered statistically significant. All statistical analyses were conducted using SPSS software (version 23.0; IBM Corp., Armonk, NY, USA). The correlation matrix was generated using GraphPad Prism (version 8.0; GraphPad Software, La Jolla, CA, USA).

## Results

### Participant characteristics

In this study, the participants were successfully induced to produce sputum. Moreover, according to sputum cell counts, 83 pediatric patients with asthma were categorized into the following subtypes: 32 eosinophilic (38.6%), 15 mixed (18.1%), 15 neutrophilic (18.1%), and 21 paucigranulocytic (25.3%). 60 asthma patients (72.3%) took daily inhaled corticosteroids (ICS). According to the categories of ICS doses by formulation and age, 42 children used low-dose ICS, and 18 children used medium-dose ICS. None of the children in the study were taking high-dose ICS.

As summarized in Table 1, no significant differences in age or sex was observed between the groups. The percentage of patients with atopy, eosinophils in induced sputum, and increased blood eosinophil count and serum total IgE levels were higher in patients with asthma than in controls. However, neutrophil counts in sputum and blood were not different between the groups.

FEV_1_, the percentage change in FEV_1_ after BD, ratio of FEV_1_ and forced vital capacity (FEV_1_/FVC), forced expiratory flow mid-expiratory phase between 25% and 75% of FVC (FEF_25–75_), and FeNO levels were significantly lower in patients with asthma than in controls. IOS parameters, including AX, R5-20, R5, R10, and X5, were significantly higher in patients with asthma than those in the controls (Table 1).

**Table 1:** Participant characteristics

Characteristics	Control (*n* = 59)	Asthma (*n* = 83)	*p*-value
Age, years	9.8 ± 2.6	8.9 ± 3.1	0.056
Sex, male (%)	37 (62.7)	58 (69.9)	0.469
Atopy, with (%)	19 (32.2)	64 (77.1)	< 0.001
Serum total IgE, IU/mL	112 (46–287)	277 (113–590)	< 0.001
Serum eosinophils, uL	180 (110–390)	440 (210–688)	< 0.001
Serum neutrophils, uL	3620 (2990–4970)	4300 (3163–6200)	0.051
Sputum eosinophils, %	0 (0–1)	4.5 (0–12.3)	< 0.001
Sputum neutrophils, %	62 (26–74)	41 (27.8–67.5)	0.225
Sputum macrophages, %	41 (23–68)	41 (17.5–62)	0.244
Spirometry parameters			
FVC, % predicted	102.8 ± 13.0	99.5 ± 12.7	0.126
FEV_1_, % predicted	106.6 ± 13.1	96.9 ± 15.1	< 0.001
FEV_1_/FVC, %	92.6 ± 9.9	85.7 ± 9.5	< 0.001
Change in FEV_1_ after BD, %	1.9 (0.6–4.5)	7.9 (2.3–12.4)	< 0.001
FEF_25-75_, % predicted	96.5 (78.7–108.9)	72 (56.1–95.3)	< 0.001
Post-BD FEV1/FVC, %	107.1 (104.8–110)	87.9 (82.3–92.2)	< 0.001
FeNO, ppb	13 (7.6–26.8)	27.5 (17.9–49.8)	< 0.001
IOS parameters			
AX, kPa/L	1.9 (1.0–2.8)	2.7 (1.6–3.8)	< 0.001
R5-20, kPa/(L/s)	0.5 ± 0.2	0.6 ± 0.2	< 0.001
R5, % predicted	87.1 (73.3–105)	98.6 (32.7–90.8)	0.002
R10, % predicted	80.5 (62.7–90.8)	85.6 (71.4–104.1)	0.014
X5, % predicted	107 (91.2–133.8)	146.1 (108.9–184.3)	< 0.001

Data are expressed as number (percentage), mean ± standard deviation, or median (interquartile range), as appropriate.

IgE, immunoglobulin E; FVC, forced expiratory vital capacity; FEV_1_, forced expiratory volume in 1 s; FEF_25 − 75_, forced expiratory flow between 25% and 75%; BD, bronchodilator; FeNO, fractional exhaled nitric oxide; IOS, impulse oscillometry; AX, reactance area; R5-20, difference between resistance at 5 and 20 Hz; R5, resistance at 5 Hz; R10, resistance at 10 Hz; X5, reactance at 5 Hz

### Sputum MPO and HNL/NGAL levels in childhood asthma

**Fig. 1:**
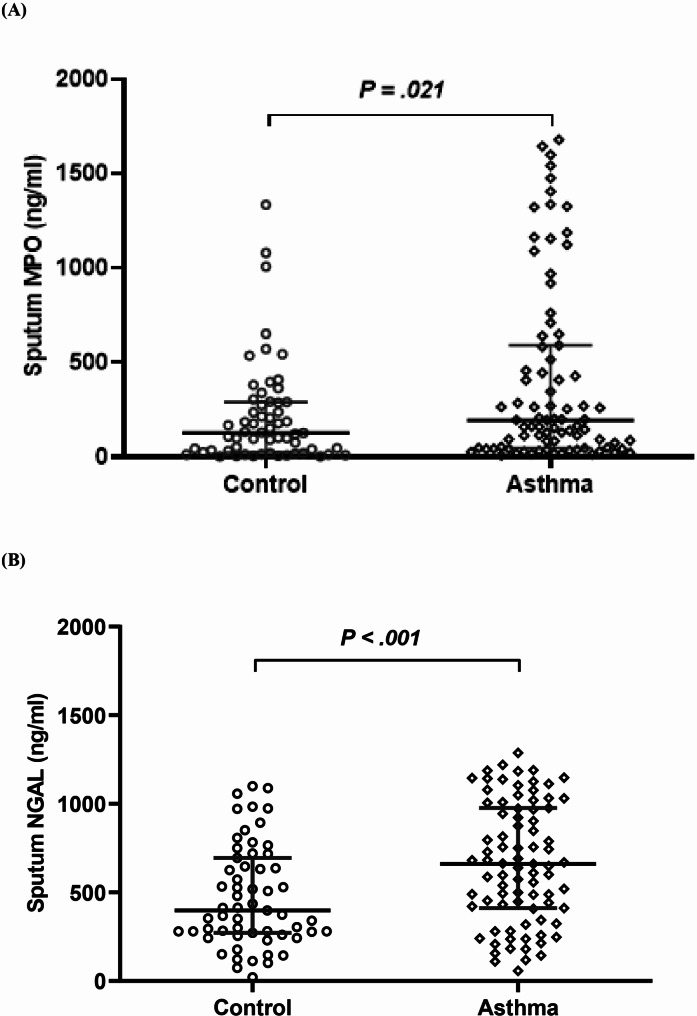
Sputum MPO and HNL/NGAL levels in participants. MPO and HNL/NGAL levels in induced sputum supernatants from healthy controls and pediatric patients with asthma. Pediatric patients with asthma had significantly higher levels of sputum MPO (**a**) and HNL/NGAL (**b**) than those of the controls. MPO, myeloperoxidase; NGAL, neutrophil gelatinase-associated lipocalin

Sputum MPO level was significantly higher in patients with asthma than in controls (194.3 [47.5–591.1] vs. 126.8 [24.3–290.8] ng/mL, *p* = 0.021) (Fig. 1a). Moreover, the expression of HNL/NGAL in the sputum was also higher in patients with asthma than in the controls (660.7 [411.6-977.1] vs. 194.3 [47.5-591.1] ng/mL, *p* < 0.001) (Fig. 1b).

**Fig. 2:**
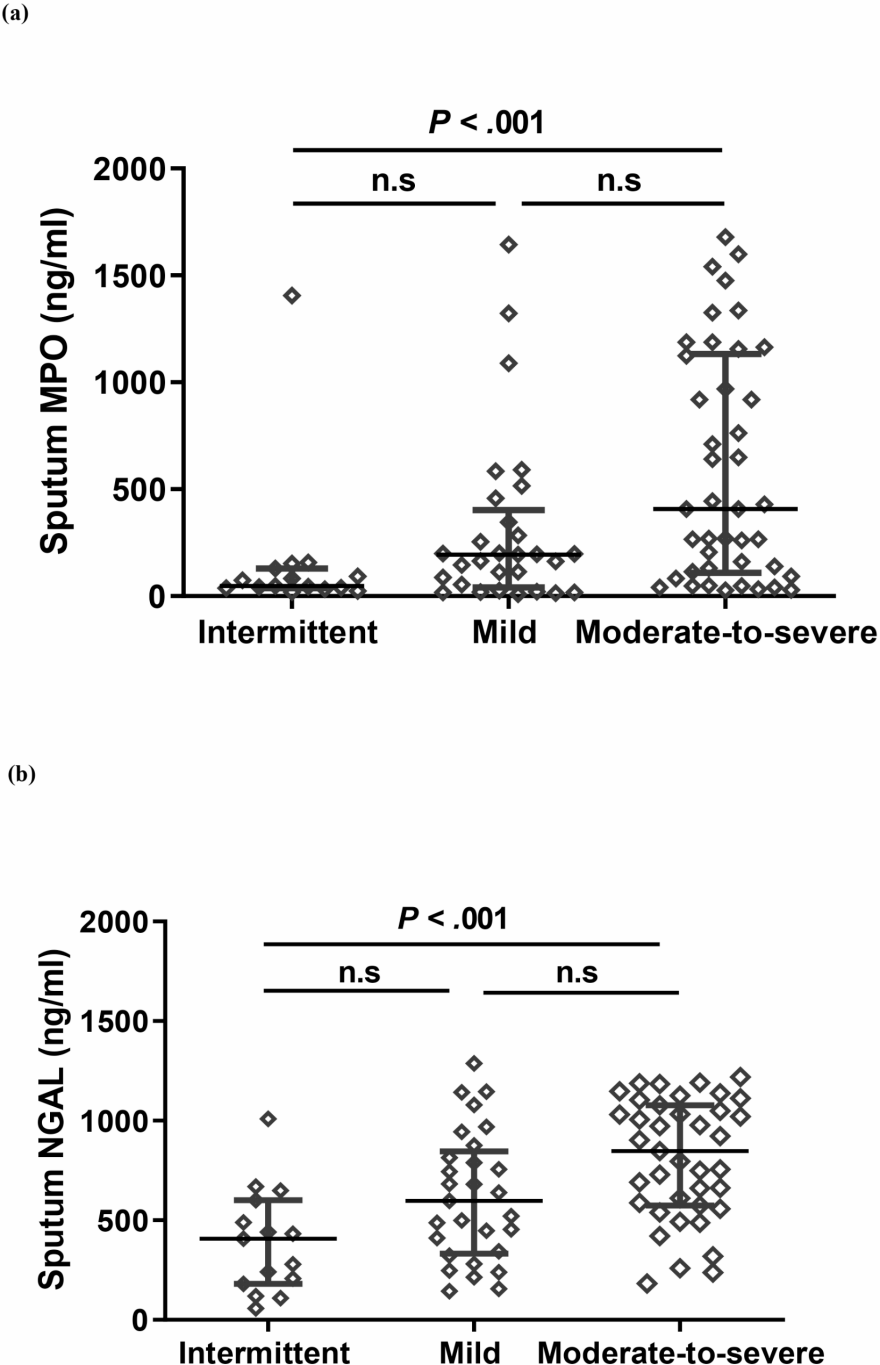
Sputum MPO and HNL/NGAL levels correlate with sputum neutrophils count in participants. (**a** and **b**) MPO and HNL/NGAL levels in sputum were positively correlated with sputum neutrophils count in all pediatric patients. (**c**) MPO and HNL/NGAL levels also exhibited positive correlation with each other. MPO, myeloperoxidase; NGAL, neutrophil gelatinase-associated lipocalin

When comparing sputum MPO and HNL/NGAL levels among the subgroups based on asthma severity (Fig. 2), patients with moderate-to-severe persistent asthma showed significantly higher sputum MPO levels than those of patients with intermittent asthma (407.6 [57.8–1,085.0] vs. 43.5 [13.2–141.4 ng/mL], *p* < 0.001). Sputum HNL/NGAL levels were also significantly higher in the moderate-to-severe persistent group than in the intermittent group (848.1 [574.1–1,077.0] vs. 408.3 [180.7–600.6] ng/mL, *p* < 0.001). The sputum MPO and HNL/NGAL levels were higher in the mild persistent group than in the intermittent asthma group; however, no statistically significant differences (*p* = 0.135 and *p* = 0.093, respectively) were observed. The sputum MPO and HNL/NGAL levels were not statistically significant between the mild and moderate-to-severe persistent groups (*p* = 0.612 and *p* = 0.065, respectively).

### Sputum MPO and HNL/NGAL levels reflecting activation of neutrophils in the airway

**Fig. 3:**
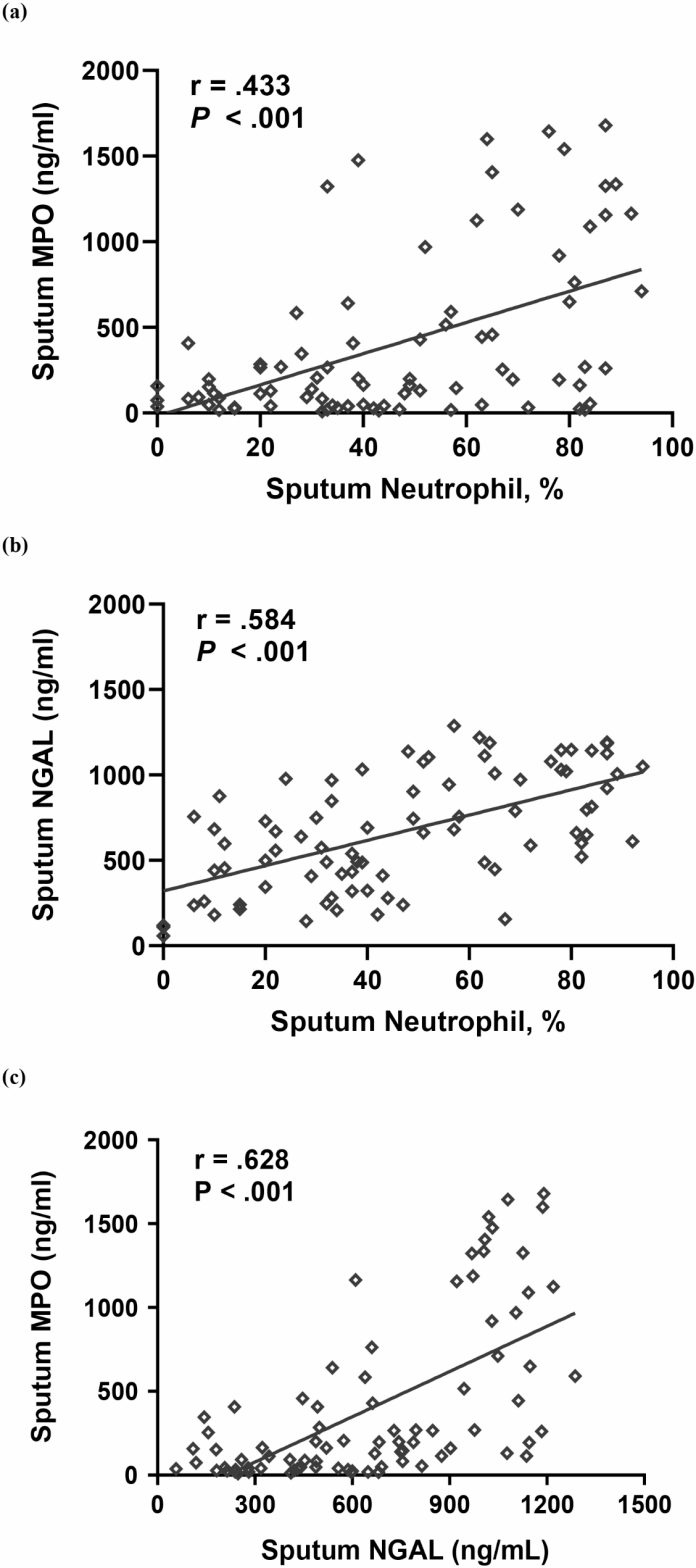
Comparison of sputum MPO and HNL/NGAL among the asthma subgroups based on severity. The sputum MPO and HNL/NGAL levels were significantly elevated in pediatric patients with moderate-to-severe asthma compared to those with intermittent asthma. MPO, myeloperoxidase; NGAL, neutrophil gelatinase-associated lipocalin

In patients with asthma, sputum MPO levels exhibited positive correlation with sputum neutrophils count (*r* = 0.433 *p* < 0.001) (Fig. 3a). Significant positive correlations were found between sputum HNL/NGAL levels and sputum neutrophils count (*r* = 0.584, *p* < 0.001) (Fig. 3b). Furthermore, sputum MPO and HNL/NGAL levels demonstrated a positive correlation with each other (*r* = 0.628, *p* < 0.001) (Fig. 3c). However, with serum neutrophils, no correlation was observed between sputum MPO or HNL/NGAL levels (Supplement 1).

### Correlation of sputum MPO and HNL/NGAL levels with pulmonary function

**Fig. 4:**
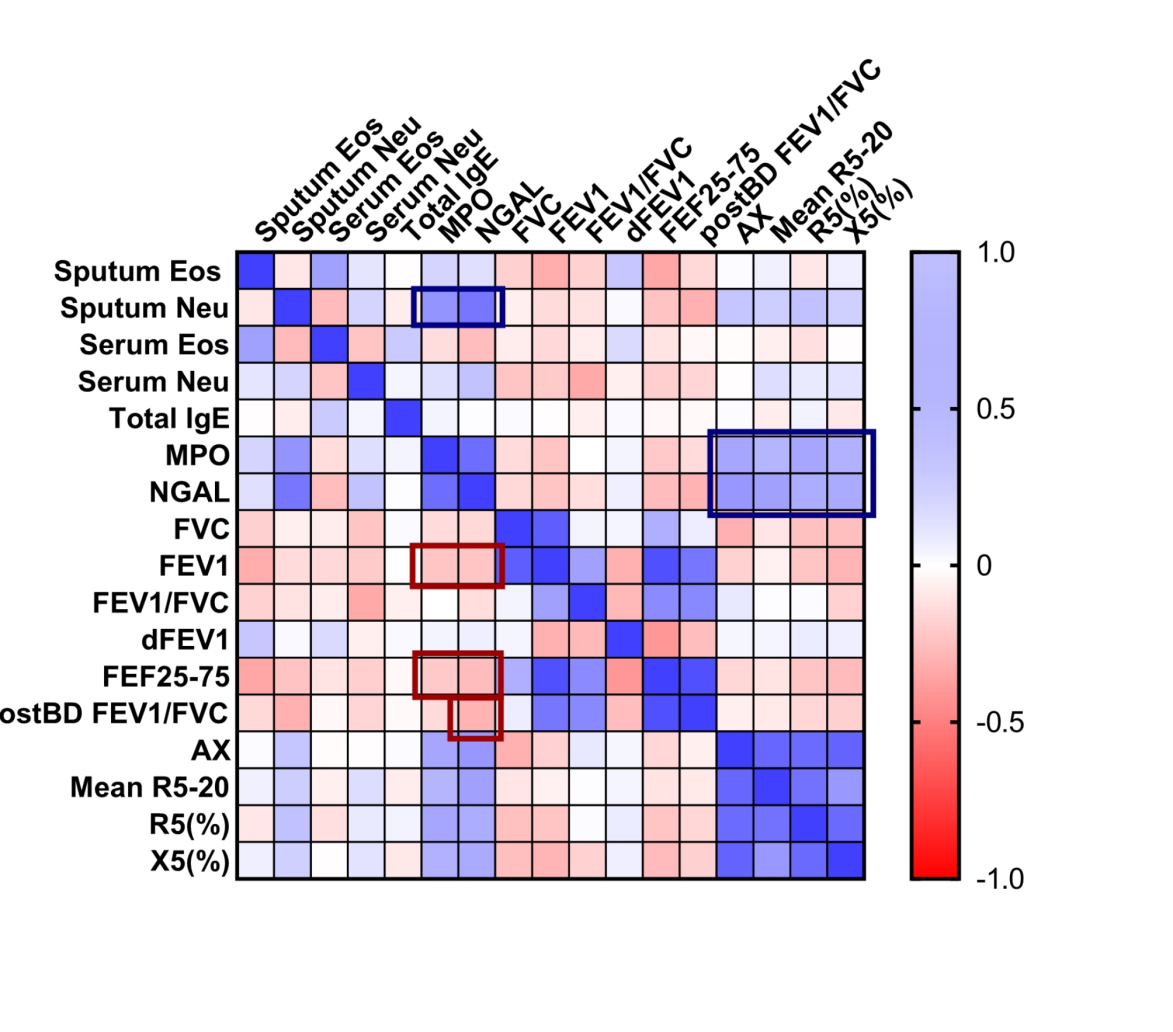
Correlation matrix. Levels of sputum neutrophil granules (MPO and HNL/NGAL) showed positive correlations with sputum neutrophils and impulse oscillometry parameters (blue boxes). Moreover, they exhibited negatively correlated with pulmonary functions (red boxes) in pediatric patients with asthma. Eos, eosinophils; Neu, neutrophils; IgE, immunoglobulin E; MPO, myeloperoxidase; NGAL, neutrophil-gelatinase associated lipocalin; FVC, forced expiratory vital capacity; FEV1, forced expiratory volume in 1 s; dFEV1, change in FEV1 after bronchodilator; FEF25-75, forced expiratory flow between 25% and 75%; BD, bronchodilator; AX, reactance area; R5-20, difference between resistance at 5 and 20 Hz; R5, resistance at 5 Hz; X5, reactance at 5 Hz

When comparing sputum MPO and HNL/NGAL levels in terms of their relationship with pulmonary function parameters, sputum MPO levels showed significant negative correlations with FEV_1_ (*r* = -0.235, *p* = 0.033), FEF_25-75_ (*r*= -0.228, *p* = 0.038). As shown in Table 2, sputum HNL/NGAL levels also exhibited significantly negative correlation with FEV_1_ (*r* = -0.233 *p* = 0.034), FEF_25-75_ (*r*= -0.265, *p* = 0.017). Sputum HNL/NGAL levels demonstrated significantly negative correlations with FEV_1_ (*r* = -0.233, *p* = 0.034), FEF_25-75_ (*r* = -0.265, *p* = 0.017), and post-BD FEV_1_ (*r* = -0.294, *p* = 0.08). Both sputum MPO and HNL/NGAL showed siginificant correlations with small airway obstructions using IOS parameters (Table 2). In this study, compared to sputum MPO level, sputum HNL/NGAL level demonstrated better ability to accurately reflect the presence of asthma and airway inflammation. However, FeNO level did not show any association with either MPO or HNL/NGAL levels in sputum. Correlations between sputum MPO level, HNL/NGAL level, and other parameters are summarized in the correlation matrix (Fig. 4).

**Table 2:** Correlation of sputum MPO and NGAL levels with pulmonary function

Spearman	Sputum MPO (ng/mL)		Sputum NGAL (ng/mL)	
	Correlation Coefficient	*p*-value	Correlation Coefficient	*p*-value
FVC_1_, % predicted	-0.143	0.198	-0.153	0.167
FEV1, % predicted	-0.235	0.033*	-0.233	0.034*
FEV_1_/FVC, %	-0.003	0.977	-0.130	0.243
Change in FEV_1_ after BD, %	0.042	0.712	0.060	0.592
FEF_25 − 75_, % predicted	-0.228	0.038*	-0.265	0.017*
Post-BD FEV_1_/FVC, %	-0.149	0.185	-0.293	0.008**
FeNO	0.190	0.073	0.132	0.216
AX, kPa/L	0.355	0.001**	0.426	< 0. 001***
R5-20, kPa/(L/s)	0.289	0.009**	0.377	0.001**
R5, % predicted	0.353	0.001**	0.328	0.003**
R10, % predicted	0.110	0.326	0.111	0.324
X5, % predicted	0.307	0.005**	0.330	0.003**

A correlation coefficient is significant at the 0.05 level.

MPO, myeloperoxidase; NGAL, neutrophil-gelatinase associated lipocalin; FVC, forced expiratory vital capacity; FEV_1_, forced expiratory volume in 1 s; FEF_25-75_, forced expiratory flow between 25% and 75%; BD, bronchodilator; FeNO, fractional exhaled nitric oxide; AX, reactance area; R5-20, difference between resistance at 5 and 20 Hz; R5, resistance at 5 Hz; R10, resistance at 10 Hz; X5, reactance at 5 Hz

## Discussion

Childhood asthma is not a single condition but rather a complex disease with various phenotypes, affected by age of onset, presence of atopy and viral wheezing episodes, or exposure to other triggers [4]. Identifying asthma phenotypes in pediatric patients helps to determine the risk factors for exacerbation, enabling the optimization of treatment strategies for preventing chronic pulmonary diseases [26]. Recent studies have investigated the spectrum of severity and phenotypic subpopulations in childhood asthma using cluster analysis, categorizing phenotypes based on biomarkers attached to endotypes for targeted therapy and prognosis prediction [1–4]. Assessing sputum inflammatory cells is essential for understanding the characteristics of asthma. These inflammatory cells, particularly eosinophils and neutrophils, are linked to asthma endotypes related to T2 or non-T2 pathways [1–4]. Quantitative sputum cytometry can aid in classifying airway inflammation patterns in asthma into eosinophilic, neutrophilic, mixed granulocytic, and paucigranulocytic subtypes [2]. However, consensus on the sputum neutrophil proportion cutoff defining neutrophilic asthma is lacking. Various cutoffs from > 40–76% neutrophils have been utilized [5]. In this study, neutrophilic inflammation was defined as sputum eosinophils < 3% and sputum neutrophils ≥ 61% [24, 25].

In this study, the noneosinophilic subgroup comprised 43.4% of patients with asthma, higher than the proportion of patients in the eosinophilic subgroup (38.6%). The levels of MPO and HNL/NGAL, secreted from azurophilic and gelatinase neutrophilic granules, were increased in pediatric patients with asthma, especially in those with moderate-to-severe persistent asthma. These levels showed positive correlations with sputum neutrophil counts but not with serum neutrophil counts. Additionally, sputum MPO and HNL/NGAL levels reflected pulmonary dysfunction in pediatric patients with asthma. Negative correlations with FEV_1_ and FEF_25 − 75_ and positive correlations with IOS parameters (AX, R5-20, R5, and X5) could support the possible association of neutrophilic inflammation with decreased pulmonary function and airway remodeling. However, sputum MPO and HNL/NGAL levels did not correlate significantly with the markers of eosinophilic inflammation, such as eosinophil counts or FeNO level. We were able to determine the correlation between sputum MPO and HNL/NGAL with other variables from the correlation matrix. Notably, our study suggests that sputum MPO and HNL/NGAL could serve as robust assessment variables for asthma severity, providing valuable insights into the current state of neutrophilic inflammation in the airways.

Neutrophils are the first line of defense against the invasion of microorganisms. They are generated in the bone marrow, recruited to the site of inflammation, and eliminate pathogens through phagocytosis or cytotoxic granules. Neutrophil granules can be divided into the following four subtypes: azurophilic (primary), specific (secondary), gelatinase (tertiary) granules, and secretory vesicles, which are synthesized. In the airways, neutrophils interact with other immune and structural cells, potentially exacerbating inflammation and airway remodeling [7, 8, 26, 27]. Previous studies have found that patients with asthma with high sputum neutrophil counts often experience decreased pulmonary function, limited airflow, and increased resistant to inhaled corticosteroids [4, 5]. Prominent neutrophilic inflammation has been found to be related to severe asthma and asthma exacerbation [2, 6, 14, 26]. Although airway neutrophils are also observed in pediatric patients with asthma, the role of neutrophils in childhood asthma is still under investigation. In our study, we did not observe an increase in sputum neutrophil counts in children with asthma. However, we did find significant differences in the activity of neutrophil granules, specifically in markers such as MPO and MPO/NGAL. This suggests that not only the number of neutrophils, but also their increased activity, may play an important role in disease activity.

Neutrophils could be recruited by proinflammatory cytokines secreted from respiratory epithelial cells and activated by T helper (Th)1 and Th17 cells. Interleukin (IL)-17 is a critical cytokine in neutrophilic asthma that regulates neutrophil migration and infiltration. Moreover, IL-17 can induce glucocorticoid receptor β on epithelial cells in patients with asthma, which may be related to glucocorticoid insensitivity in neutrophilic asthma [26, 28]. Elevated mediators and cytokines, such as leukotriene B4, tumor necrosis factor α, IL-8, and IL-17, in neutrophilic asthma may induce migration and proliferation of airway smooth muscle cells, resulting in airway hyperresponsiveness and remodeling [26, 28, 29]. Even in severe asthma, corticosteroid treatment may reduce the apoptosis of neutrophils in asthma. Delayed apoptosis of neutrophils due to epithelial cell-driven neutrophil chemotactic and antiapoptotic activity may induce impaired macrophage efferocytosis and an altered airway microbiome. Neutrophil apoptosis defects cause an excessive inflammatory response [5, 6]. Activated neutrophils can cause airway hyperresponsiveness, bronchospasm, airway stenosis, lung tissue damage, mucus gland hyperplasia, hypersecretion, and airway remodeling. These changes can lead to permanent alterations in the airway structure.

Despite advancements in the treatment of asthma, many patients, particularly those with neutrophilic asthma, do not experience significant improvement [27, 28]. Moreover, T2-low asthma is still difficult to diagnose because of a lack of appropriate biomarkers [14, 26, 28]. To alleviate the symptoms and reduce the frequency of acute exacerbations in these patients, targeted therapy for neutrophil-dominated inflammation is required. Clinical trials targeting neutrophil elastase have shown improved pulmonary function in patients with cystic fibrosis and non-cystic fibrosis bronchiectasis [29]. Targeting neutrophilic inflammation is crucial for controlling inflammation, especially in cases of moderate-to-severe persistent asthmaFurther research is necessary to explore the specific mechanisms by which neutrophils contribute to the onset and progression of asthma. Understanding the role of neutrophils in asthma may lead to personalized therapeutic interventions [26–30].

This study has some limitations. The cross-sectional and retrospective design prevented the establishment of the cause-and-effect relationships. We measured IL-8 and IL-17 levels in the sputum in relation to Th1 and Th17, respectively. However, they showed deficient concentrations, and thus we could not obtain a statistically significant result. It was also difficult to compare the responses of patients to steroid treatment, partly due to the small sample size in each subgroup. In addition, we excluded diverse clinical conditions affecting neutrophil activation, such as acute infections, duration of illness, or other chronic inflammatory diseases. However, we could not exclude environmental exposures, such as passive smoking and heavy pollutant exposure or accompanying treatments, which could also affect neutrophil granule expression [28]. To address these limitations, subsequent prospective research with a large sample size and long follow-up period is necessary to clarify the role of neutrophils in the development of specific subtypes of asthma and the response to treatment.

## Conclusions

Our study advances the understanding of neutrophilic inflammation in childhood asthma, proposing MPO and HNL/NGAL as potential markers for assessing asthma severity. These findings may contribute to the ongoing efforts to unravel the heterogeneity of asthma phenotypes and hold promise for personalized approaches in managing this complex respiratory condition.

## Electronic supplementary material

Below is the link to the electronic supplementary material.

**Supplementary Material 1**: **Supplement 1**. Correlation of Sputum MPO and HNL/NGAL levels and serum neutrophils. Supplement 1 showed no correlations between sputum MPO or HNL/NGAL levels with serum neutrophils

**Supplementary Material 2**: **Supplement 2**. Correlation of Sputum MPO and HNL/NGAL levels and pulmonary functions. Supplement 2 showed correlations between sputum MPO or HNL/NGAL levels with pulmonary function parameters in graphs

## Data Availability

The datasets used and/or analyzed during the current study are available from the corresponding author on reasonable request. However, due to ethical restrictions (regulation by Institutional Review Board), they are not publicly available.

## Abbreviations

AX: Reactance area
BD: Bronchodilator
BHR: Bronchial hyperresponsiveness
FEF_25 − 75_: Forced expiratory flow mid-expiratory phase between 25% and 75% of FVC
FeNO: Fractional concentration of exhaled nitric oxide
FEV_1_: Forced expiratory volume in 1 s
FVC: Forced vital capacity
HNL/NGAL: Human neutrophil lipocalin or neutrophil gelatinase-associated lipocalin
ICS: Inhaled corticosteroids
IgE: Immunoglobulin E
IL: Interleukin
IOS: Impulse oscillometry
MPO: Myeloperoxidase
R5: 5 Hz
R10: 10 Hz
R5-20: Difference between respiratory resistance at 5 and 20 Hz
T2: Type 2 inflammation
Th: T helper
X5: Reactance values at 5 Hz
