# Supplementary material for: Release of sputum neutrophil granules is associated with pulmonary function and disease severity in childhood asthma
Source: BMC Pulm Med. 2024 Oct 24;24:532. doi: 10.1186/s12890-024-03340-y (PMC11515414; doi:10.1186/s12890-024-03340-y)
Supplement: Supplementary file 1 — Supplementary Material 1: Supplement 1. Correlation of Sputum MPO and HNL/NGAL levels and serum neutrophils. Supplement 1 showed no correlations between sputum MPO or HNL/NGAL levels with serum neutrophils [file 12890_2024_3340_MOESM1_ESM.docx]

**Additional File 1.** Correlation of Sputum MPO and HNL/NGAL levels and serum neutrophils

**
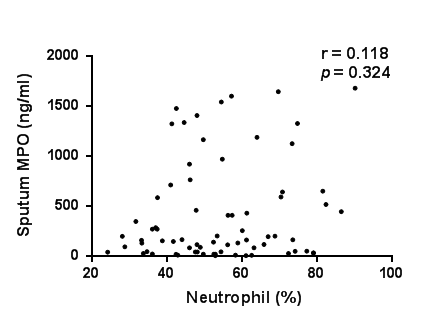
(a)**


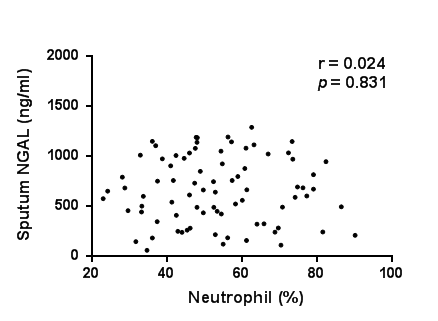
**(b)**

Regarding neutrophils in the blood, no association was observed between sputum MPO and HNL/NGAL levels and serum neutrophils.

MPO, myeloperoxidase; NGAL, neutrophil gelatinase-associated lipocalin
