# Supplementary material for: Release of sputum neutrophil granules is associated with pulmonary function and disease severity in childhood asthma
Source: BMC Pulm Med. 2024 Oct 24;24:532. doi: 10.1186/s12890-024-03340-y (PMC11515414; doi:10.1186/s12890-024-03340-y)
Supplement: Supplementary file 2 — Supplementary Material 2: Supplement 2. Correlation of Sputum MPO and HNL/NGAL levels and pulmonary functions. Supplement 2 showed correlations between sputum MPO or HNL/NGAL levels with pulmonary function parameters in graphs [file 12890_2024_3340_MOESM2_ESM.docx]

**Additional File 2.** Correlation of Sputum MPO and HNL/NGAL levels and pulmonary functions.

**(a)**

**(b)**

**(c)**

**(d)**

**(e)**

**(f)**

The meaningful correlations found in Table 2 were represented graphically.

MPO, myeloperoxidase; NGAL, neutrophil gelatinase-associated lipocalin; FEV_1_, forced expiratory volume in 1 s; FEF_25-75_, forced expiratory flow between 25% and 75%; AX, reactance area; R5-20, difference between resistance at 5 and 20 Hz; R5, resistance at 5 Hz; X5, reactance at 5 Hz.
